# Supplementary material for: A double-edged hashtag: Evaluation of #ADHD-related TikTok content and its associations with perceptions of ADHD
Source: PLoS One. 2025 Mar 19;20(3):e0319335. doi: 10.1371/journal.pone.0319335 (PMC11922258; doi:10.1371/journal.pone.0319335)
Supplement: S1 Appendix — (DOCX) [file pone.0319335.s001.docx]

**Appendix A**

**Table A1**

*Descriptive Statistics of Study Variables for Non-Binary, Two-Spirit, Agender, Questioning, and Participants Who Chose not to Disclose Their Gender Identity* (*N* = 49)

|  | Total  *M*(*SD*) |
| --- | --- |
| Confidence in own ADHD |  |
| T1 | 5.45 (1.46) |
| T2 | 5.30 (1.61) |
| T3 | 4.13 (0.71) |
| ASRS |  |
| ASRS-with | 3.70 (0.49) |
| ASRS-without | 2.88 (0.89) |
| ADHD-TikTok consumption | 2.54 (0.84) |
| Perception of typical content | 3.15 (1.00) |
| Recommend typical content | 3.27 (1.23) |
| Evaluation top 5 videos | 3.44 (0.76) |
| Evaluation bottom 5 videos | 2.57 (0.54) |
| Recommendation top 5 videos | 2.69 (0.83) |
| Recommendation bottom 5 videos | 2.18 (0.66) |
| Estimated ADHD prevalence |  |
| General population | 26.08 (17.63) |
| Family members | 41.88 (28.22) |
| Friends | 50.14 (30.25) |
| Watch psychologist video (% yes) | 65.3% |
